# Metabolic engineering of *Escherichia coli* BW25113 for the production of 5-Aminolevulinic Acid based on CRISPR/Cas9 mediated gene knockout and metabolic pathway modification

**DOI:** 10.1186/s13036-022-00307-7

**Published:** 2022-10-13

**Authors:** Changchuan Ye, Yuting Yang, Xi Chen, Lijie Yang, Xia Hua, Mengjie Yang, Xiangfang Zeng, Shiyan Qiao

**Affiliations:** 1 State Key Laboratory of Animal Nutrition, Ministry of Agriculture Feed Industry Centre, China Agricultural University, Beijing, 100193 China; 2 Beijing Key Laboratory of Bio-Feed Additives, Beijing, 100193 China; 3 State Key Laboratory for Agro-Biotechnology, and Ministry of Agriculture and Rural Affairs, Key Laboratory for Pest Monitoring and Green Management, Department of Plant Pathology, China Agricultural University, Beijing, 100193 China; 4 National Feed Engineering Technology Research Centre, Beijing, 100193 China

**Keywords:** *E. coli*, 5-Aminolevulinic Acid, CRISPR/cas9, T7 Expression System, Metabolic engineering

## Abstract

**Background:**

5-Aminolevulinic acid (ALA) recently received much attention due to its potential application in many fields. In this study, an ALA production strain of *Escherichia coli* was constructed by rational metabolic engineering and stepwise improvement based on known regulatory and metabolic information and CRISPR/Cas9 mediated gene knockout.

**Results:**

A metabolic strategy to produce ALA directly from glucose in this recombinant *E. coli* via the C5 pathway was applied herein. The rational metabolic engineering by gene knockouts significantly improved ALA production from 662.3 to 1601.7 mg/L. In addition, we managed to synergistically produce ALA via the C4 pathway in recombinant strain. The expression of a modified *hemA* gene, encoding an ALA synthase from *Rhodobacter sphaeroides*, improved ALA production from 1601.7 to 2099.7 mg/L. After 24 h cultivation, a yield of 0.210 g ALA per g glucose was achieved by constructed *E. coli* D5:FYABD-RSA.

**Conclusion:**

Our study revealed that an industrially competitive strain can be efficiently developed by metabolic engineering based on combined rational modification and optimization of gene expression.

**Supplementary Information:**

The online version contains supplementary material available at 10.1186/s13036-022-00307-7.

## Background

Synthetic biology plays a critical part in bio-based production of fuels, chemicals and materials from biomass. The sophistication of available genetic and biochemical tools has developed as synthetic biology applications have grown in complexity. In the past 20 years, a dramatic change in the scope and complexity of efforts within the space of synthetic biology has occurred [1]. Due to the great development of genetic engineering, accomplishments have progressed from simple reconstitution of biosynthetic pathways in heterologous hosts to complicated refactoring efforts [2–4] that can produce medicinally relevant compounds (strictosamide, taxadiene, etc.) [5, 6] or amino acids (L-valine, L-threonine, etc.) [7, 8] in a high titer.

Among many systems available for heterologous protein production, the Gram-negative bacterium *Escherichia coli* remains one of the most attractive organisms because of its ability to grow rapidly and at high density on inexpensive substrates. Well-characterized genetics and availability of a large number of cloning vectors and mutant host strains [9]. As a commonly used organism for expression and optimization of heterologous biosynthetic pathways, *E. coli* has been the most widely used prokaryotic system that produces heterologous proteins for industrial production of bacterial metabolites by batch and fed-batch operations [10, 11]. *E. coli* is a facultative anaerobic, non-sporulating bacterium that is considered as the workhorse of modern biotechnology in the microbial production of bio-fuels and biochemicals [12]. Recent advances in the fundamental understanding of transcription, translation and protein folding in *E. coli*, together with developments of improved genetic tools have made *E. coli* more valuable than ever [9].

5-Aminolevulinic acid (ALA) is a key intermediate involved in the biosynthesis of tetrapyrrole, which has attracted much attention for its potential applications in agriculture, cosmetics and cancer therapy. Due to its nontoxicity to crops, animals and humans, ALA is also used as selective biodegradable herbicide and insecticide in agriculture [13].

In living organisms, there are two major pathways described for ALA biosynthesis [13]. One is the C4 pathway, which occurs in mammals, birds, yeast and purple non-sulfur photosynthetic bacteria. In this pathway, ALA is formed through catalyzation of 5-aminolevulinate synthase, which condenses glycine and succinyl-CoA, an intermediate of the tricarboxylic acid (TCA) cycle [14]. The second pathway, C5, is present in higher plants, algae and many bacteria including *E. coli* [15]. In the C5 pathway, glutamate is the only substrate for biosynthesis of ALA.

In *E. coli*, ALA biosynthesis is through the C5 pathway and is tightly regulated by feedback inhibition of heme, the end product of the C5 pathway [16]. Earlier study has developed an available metabolic strategy of ALA biosynthesis via C5 pathway in *E. coli* [17]. Through this strategy, ALA can be synthesized in *E. coli* by over-expressing the key genes, *hemA* and *hemL*, in a modified minimal medium using glucose as the sole carbon source. In the research reported herein, we used a similar process for ALA biosynthesis. We managed to integrate *T7 RNA Polymerase* into the genome of *E. coli* BW25113 in our earlier work. This mutant strain was named *E. coli* BW25113-T7 [18]. In this mutant strain, the key genes, *hemA*, *hemL* and *RhtA,* were expressed efficiently and controlled by the T7 Expression System.

Our earlier work proved that this process for ALA biosynthesis in *E. coli* BW25113-T7 works. Through efficient and controllable protein expression using the T7-Lac promoter, *E. coli* BW25113-T7 accumulated 42.4% more ALA than *E. coli* BL21(DE3) did [18]. Here, we report the development of the genetically defined ALA overproducing *E. coli* strain by systems metabolic engineering. It was thus our goal to engineer *E. coli* to make a 5-Aminolevulinic acid overproducer with completely known genotype by systems-level metabolic engineering. In this study, rational metabolic engineering based on known regulatory and metabolic information was carried out to develop an *E. coli* strain capable of overproducing ALA.

## Results

### Available metabolic strategy of ALA production by overexpression of *hemA*,* hemL* and *eamA*

**Fig. 1:**
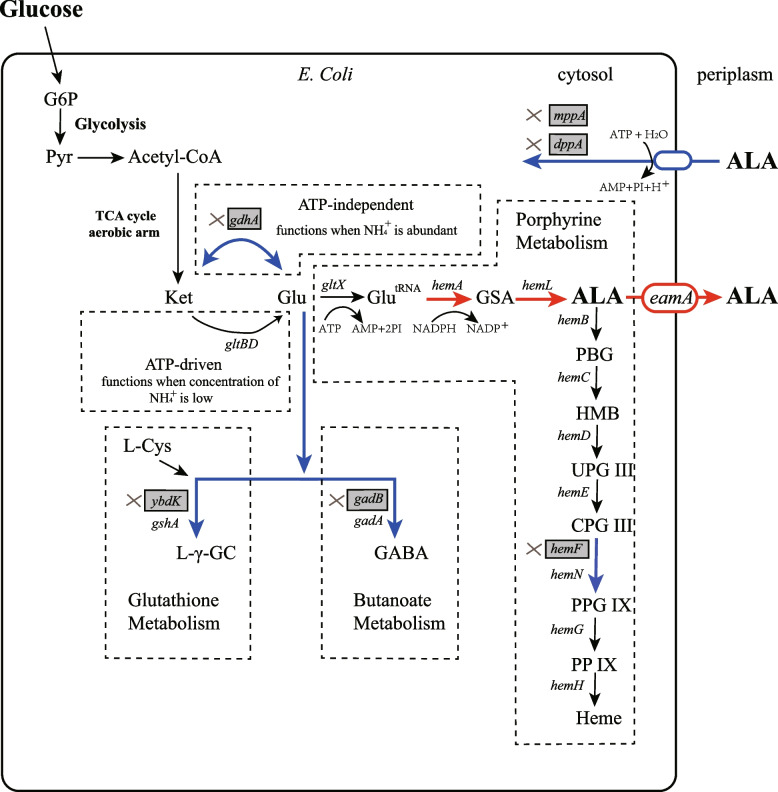
The biosynthetic pathways of ALA in *E. coli* and the strategies for constructing the ALA producing strain. The shaded boxes indicate the genes that were knocked out. Red arrows indicate increased flux or activity by directly over-expressing the corresponding genes. Blue arrows indicate decreased flux or activity by knocking out the corresponding genes. Dotted boxes represent the corresponding metabolic pathways

We constructed an *E. coli* strain from BW25113 which achieved high efficiency of protein expression through T7 expression system in our earlier work [18]. This mutant *E. coli* BW25113-T7 strain was rationally engineered to produce ALA by over-expression of key genes. The biosynthetic pathways of ALA in *E. coli* and the strategies for constructing ALA production strain are shown in Fig. 1. The key genes of ALA synthesis via the C5 pathway in this strategy are *hemA* and *hemL*. The *hemA* gene encodes a NADPH-dependent glutamyl-tRNA reductase which catalyzes the reduction of glutamyl-tRNA to glutamate-1-semialdehyde (GSA) [17]. The *hemL* gene encodes glutamate-1-semialdehyde aminotransferase which quickly converts GSA to ALA. The *eamA* gene encodes an O-acetylserine or cysteine exporter which is capable of translocating dipeptides and amino acid analogs from the cytosol to the periplasm.

Plasmid pET-ALA-LAA was constructed to over-express these three key genes. In this plasmid, these three genes can be tightly controlled and efficiently expressed by the T7-Lac promoter. Cells that harbored pET-ALA-LAA were cultured in M9YE medium. After 24 h of induced fermentation, an ALA titer (662.3 mg/L) was determined.

### Overexpression of *gltB*,* gltD* and *gltX* genes involved in the C5 pathway in* E. coli*

Glutamate and L-glutamyl-tRNA are both precursors of ALA synthesis via the C5 pathway. To increase glutamate and L-glutamyl-tRNA amounts for ALA synthesis, we overexpressed *gltB*, *gltD* and *gltX* genes alone or in combination.

**Fig. 2:**
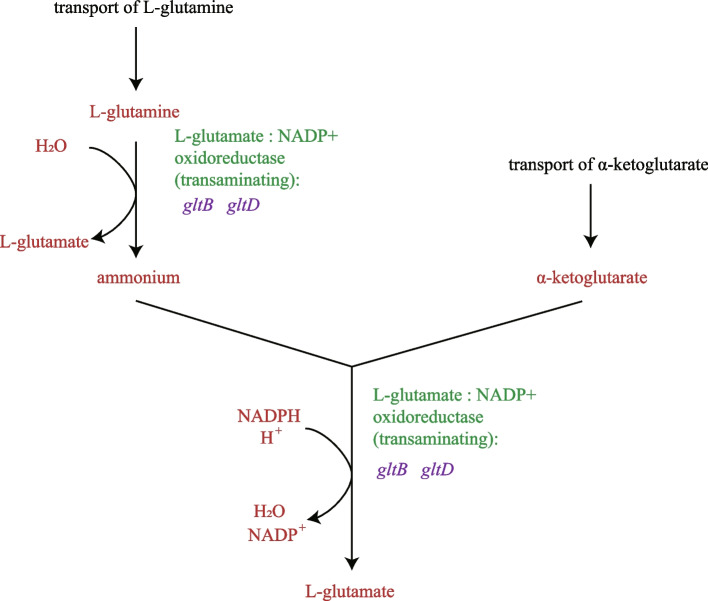
The ATP-driven pathway of L-glutamate biosynthesis in *E. coli*

There are two pathways by which *E. coli* synthesizes glutamate from ammonia [19]. The pathway catalyzed by glutamate dehydrogenase (encoded by *gdhA*) is ATP-independent. The other is a cyclic and ATP-driven pathway (Shown in Fig. 2) which catalyzed by glutamate synthase (encoded by *gltBD*). Our modified minimal medium does not provide a high level of ammonia. In this case, the ATP-driven pathway functions (Fig. 1). Glutamate synthase is a tetramer of dimers, with each dimer having one large and one small subunit (*gltB* and *gltD*, respectively) [19]. Glutamate synthase catalyzes the single-step conversion of L-glutamine and alpha-ketoglutarate into two molecules of L-glutamate (Fig. 2). In doing so, glutamate synthase simultaneously operates as the major source of L-glutamate for the cell and as a key step in ammonia assimilation during nitrogen-limited growth [19–21]. The ammonia-dependent activity can be catalyzed very slowly by just the small subunit in the absence of the full complex [22]. Glutamate-tRNA ligase (GluRS) is a member of the family of aminoacyl-tRNA synthetases, which is encoded by *gltX* gene [23, 24]. GluRS charges Glu^tRNA^ for both protein and ALA synthesis [25].

**Fig. 3:**
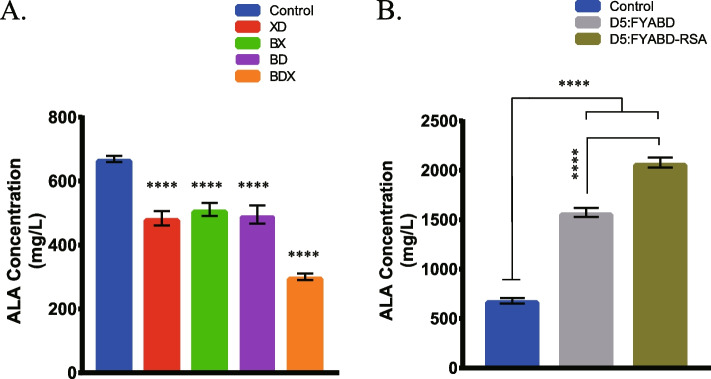
ALA production in recombinant *E. coli* expressing various related genes. Cultivation was performed in 300 mL Erlenmeyer flask supplied with 30 mL modified minimal medium supplied with 10 g/L glucose for 24 h. Results are the average of three individual experiments. A final ALA accumulation result was measured. Adjusted *P* values were calculated using Dunnett's multiple comparisons test (*P < 0.05, **P < 0.01, ***P < 0.001, ****P < 0.0001)

In this study, we constructed four extra plasmids to overexpress these three genes (Table 1). These four plasmids were electroporated into cells that harbored pET-ALA-LAA. The resulting strains were cultivated in modified minimal medium (see ‘‘Section Materials and Methods’’) supplied with 10 g/L glucose for ALA accumulation analysis. After 24 h of fermentation, ALA concentration was determined (Table 2 and Fig. 3A). Strain BW25113-T7 which only contained pET-ALA-LAA was set as the control.

**Table 1:** Strains and plasmids employed in this study

Strain or plasmid	Relevant characteristic(s)	Source and/or reference
Strain		
*E. coli* DH5α	*F-,φ80dlacZ ΔM15,Δ(lacZYA -argF)U169, deoR, recA1, endA1,hsdR17 (rK-, mK* +*), phoA, supE44, λ-, thi-1, gyrA96, rel*	TransGen, Beijing
*E. coli* BW25113	*F-, DE(araD-araB)567, lacZ4787(del)::rrnB-3, LAM-, rph-1, DE(rhaD-rhaB)568, hsdR514*	Laboratory stock
*E. coli* BW25113-T7 (D0)	BW25113 *int::(lacI::PlacUV5::T7 gene) ΔybhC*	Laboratory stock
*E. coli* D1:F	BW25113-T7 *ΔhemF*	This study
*E. coli* D1:D	BW25113-T7 *ΔdppA*	This study
*E. coli* D1:M	BW25113-T7 *ΔmppA*	This study
*E. coli* D2:FY	BW25113-T7 *ΔhemF, ΔybdK*	This study
*E. coli* D2:FA	BW25113-T7 *ΔhemF, ΔgdhA*	This study
*E. coli* D2:FB	BW25113-T7 *ΔhemF, ΔgadB*	This study
*E. coli* D3:FYA	BW25113-T7 *ΔhemF, ΔybdK, ΔgdhA*	This study
*E. coli* D3:FYB	BW25113-T7 *ΔhemF, ΔybdK, ΔgadB*	This study
*E. coli* D3:FAB	BW25113-T7 *ΔhemF, ΔgdhA, ΔgadB*	This study
*E. coli* D4:FYAB	BW25113-T7 *ΔhemF, ΔybdK, ΔgdhA, ΔgadB*	This study
*E. coli* D5:FYABD	BW25113-T7 *ΔhemF, ΔybdK, ΔgdhA, ΔgadB*, *ΔdppA*	This study
*E. coli* D5:FYABM	BW25113-T7 *ΔhemF, ΔybdK, ΔgdhA, ΔgadB*, *ΔmppA*	This study
*E. coli* D6:FYABMD	BW25113-T7 *ΔhemF, ΔybdK, ΔgdhA, ΔgadB, ΔmppA, ΔdppA*	This study
Plasmids		
pUC57	Cloning vector, AmpR, ColE1/pMB1/pBR322/pUC ori	Laboratory stock
pACYCD	Cloning vector, CmR, p15a ori	Laboratory stock
pET28b-ALA-LAA	plasmid for biosyntheizing ALA (f1 ori; KanR; *hemA*; *hemL*; *eamA*; *LacI* gene and T7-LacI promoter)	Laboratory stock
pUC-gltX-gltD	pUC57 containing *gltX* and *gltD* genes; J23107 promoter	This study
pUC-gltB-gltX	pUC57 containing *gltB* and *gltX* genes; J23107 promoter	This study
pUC-gltB-gltD	pUC57 containing *gltB* and *gltD* genes; J23107 promoter	This study
pUC-gltB-gltD-gltX	pUC57 containing *gltB, gltD* and *gltX* genes; J23107 promoter	This study
pCas	plasmid for CRISPR (temperature sensitive oriR101; KanR; the λ-Red operon under the control of arabinose-inducible promoter; *S. pyogenes*-derived cas9; sgRNA guided to ori-p15a under the control of lac operator)	Laboratory stock
pTarget-gene	plasmid for CRISPR (p15a ori; CmR; sgRNA guided to targeted gene, such as *hemF*, *ybdK*, *gdhA*, *gadB*, *dppA* or *mppA* ge	This study
pACYCD-gene	pACYCD containing targeted gene (such as *hemF*, *ybdK*, *gdhA*, *gadB*, *dppA* or *mppA*)	This study
pACYCD-Donor DNA	pACYCD containing Donor DNA for targeted gene (such as *hemF*, *ybdK*, *gdhA*, *gadB*, *dppA* or *mppA*)	This study
pACYCD-RS-hemA	pACYCD containing *hemA* from *R. sphaeroides* (amino acid sequence has been modified); T7-LacI promoter	This study

**Table 2:** ALA production in recombinant *E. coli* expressing various related genes

Strain	Relevant characteristic(s)	Plasmid	Expressed genes	ALA accumulation (mg/L)	Standard Error	Relative Change	ALA production rate (g/g)
*E. coli* BW25113-T7	BW25113-T7	pET-ALA-LAA	*hemA, hemL, eamA*	662.25	100.28	1.00	0.066
*E. coli* XD	BW25113-T7	pET-ALA-LAA + pUC-gltX-gltD	*hemA, hemL, eamA, gtlX, gltD*	476.37	18.45	0.72	0.048
*E. coli* BX	BW25113-T7	pET-ALA-LAA + pUC-gltB-gltX	*hemA, hemL, eamA, gltB, gltX*	503.16	37.35	0.76	0.050
*E. coli* BD	BW25113-T7	pET-ALA-LAA + pUC-gltB-gltD	*hemA, hemL, eamA, gltB, gltD*	487.92	46.40	0.74	0.049
*E. coli* BDX	BW25113-T7	pET-ALA-LAA + pUC-gltB-gltX-gltD	*hemA, hemL, eamA, gltB, gltD, gltX*	293.65	74.89	0.44	0.029
*E. coli* D5:FYABD	BW25113-T7 *ΔhemF, ΔybdK, ΔgdhA, ΔgadB, ΔdppA*	pET-ALA-LAA	*hemA, hemL, eamA*	1601.72	98.70	2.42	0.160
*E. coli* D5:FYABD-RSA	BW25113-T7 *ΔhemF, ΔybdK, ΔgdhA, ΔgadB, ΔdppA*	pET-ALA-LAA + pACYCD-RS-hemA	*hemA, hemL, eamA, hemA(RS)*	2099.71	159.20	3.17	0.210

A 1% (v/v) inoculum from an overnight culture for 12 h was used. IPTG was added when OD600 reached 0.7. Samples were taken and measured until 24 h. 10 g/L glucose was added initially as sole carbon source. Glycine (2 g/L) as substrate for C4 pathway was added as indicated. The results are the average of three individual experiments. ALA production rate: yield of ALA (g) per g glucose

Beyond our expectation, we found all strains that harbored extra plasmids exhibited decreased ALA accumulation with *E. coli* BDX having the greatest decrease in 5-ALA accumulation relative to the control (Table 2). We found that all strains harbored extra plasmids grew much more slowly than control strains (Table S1). These results indicate that overexpression of *gltB*, *gltD* and *gltX* genes may not have a positive effect on the ALA biosynthesis in *E. coli*. This finding suggests that the biosynthesis of L-glutamate and conversion of L-glutamate to Glu^tRNA^ are not rate-limiting steps of ALA biosynthesis.

### Enhanced production of 5-ALA by metabolic pathway modification

To further improve the BW25113-T7 strain, following targeted genetic modifications were performed (Fig. 1).

*HemF* is a strictly aerobic enzyme that requires molecular oxygen as the electron acceptor and produces hydrogen peroxide. The knock-out of *hemF* gene would mightily repress the biosynthesis of protoporphyrinogen IX (PPG) resulting in reduced endogenous loss of 5-ALA. After knocking out the *hemF* gene in BW25113-T7 (named D1:F), yield of 5-ALA increased 2.06-fold compared to the original strain (named as D0). This result indicated that knock-out of the *hemF* gene has a positive effect on ALA biosynthesis.

**Fig. 4:**
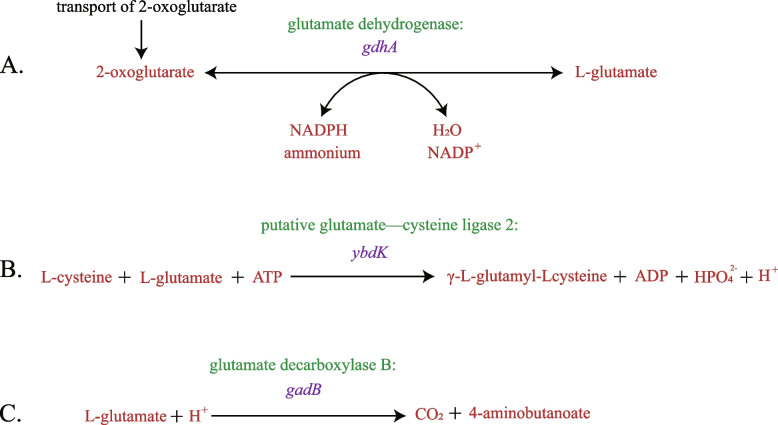
The reactions catalyzed by corresponding genes. The reaction directions shown in accordance with the physiological direction of the reaction. **A** *GdhA* catalyzes the ATP-independent amination of α-ketoglutarate to yield L-glutamate. **B** *YbdK* catalyzes the ATP-dependent ligation of glutamate with cysteine. **C** *GadB* catalyzes the cleavage of L-glutamate into carbon dioxide and 4-aminobutanoate

L-glutamate is a precursor of ALA synthesis via the C5 pathway. Therefore, we attempted to increase ALA accumulation by reducing endogenous loss of L-glutamate. *GdhA*, which encodes glutamate dehydrogenase, catalyzes the ATP-independent amination of α-ketoglutarate to yield L-glutamate [26]. This reaction is reversible (Fig. 4A). *YbdK* catalyzes ATP-dependent ligation of glutamate with cysteine at a low catalytic rate [27] (Fig. 4B). *GadB*, a glutamate decarboxylase enzyme, catalyzes cleavage of L-glutamate into carbon dioxide and 4-aminobutanoate [28] (Fig. 4C). These three genes in the ALA biosynthetic pathway were knocked out (Fig. 1) individually or jointly using the *E. coli* D1:F strain. ALA accumulations of those mutant strains ranged from a reduction to a 2.30-fold increase in ALA accumulation (Table 3). Among the mutant strains, *E. coli* D3:FYB significantly increased ALA accumulation (2.30-fold) compared with *E. coli* D0.

**Table 3:** ALA accumulation of mutant strains

Strain	genes knock-out	ALA accumulation (mg/L)	Standard Error	Relative Change	ALA production rate (g/g)	Significant Summary	*P* value
*E. coli* BW25113-T7 (D0)	Control	662.25	100.28	1.00	0.066		
*E. coli* D1:F	*ΔhemF*	1361.22	100.90	2.06	0.136	****	< 0.0001
*E. coli* D1:D	*ΔdppA*	847.07	39.61	1.28	0.085	ns	0.4304
*E. coli* D1:M	*ΔmppA*	601.37	41.40	0.91	0.060	ns	0.9991
*E. coli* D2:FY	*ΔhemF, ΔybdK*	1280.62	207.11	1.93	0.128	****	< 0.0001
*E. coli* D2:FA	*ΔhemF, ΔgdhA*	1114.22	84.14	1.68	0.111	***	0.0008
*E. coli* D2:FB	*ΔhemF, ΔgadB*	1094.72	156.11	1.65	0.109	**	0.0014
*E. coli* D3:FYA	*ΔhemF, ΔybdK, ΔgdhA*	1473.67	18.11	2.23	0.147	****	< 0.0001
*E. coli* D3:FYB	*ΔhemF, ΔybdK, ΔgadB*	1526.32	25.79	2.30	0.153	****	< 0.0001
*E. coli* D3:FAB	*ΔhemF, ΔgdhA, ΔgadB*	1341.72	274.49	2.03	0.134	****	< 0.0001
*E. coli* D4:FYAB	*ΔhemF, ΔybdK, ΔgdhA, ΔgadB*	1434.02	137.53	2.17	0.143	****	< 0.0001
*E. coli* D5:FYABD	*ΔhemF, ΔybdK, ΔgdhA, ΔgadB, ΔmppA*	1601.72	40.62	2.42	0.160	****	< 0.0001
*E. coli* D5:FYABM	*ΔhemF, ΔybdK, ΔgdhA, ΔgadB, ΔdppA*	1278.67	161.32	1.93	0.128	****	< 0.0001
*E. coli* D6:FYABMD	*ΔhemF, ΔybdK, ΔgdhA, ΔgadB, ΔmppA, ΔdppA*	1014.77	122.83	1.53	0.101	*	0.0124

A 1% (v/v) inoculum from an overnight culture for 12 h was used. IPTG was added when OD600 reached 0.7. ALA production rate: yield of ALA (g) per g glucose. Samples were collected and measured until 24 h. Glucose (10 g/L) was added initially as the sole carbon source. Results are the average of three individual replications. *P* values were calculated using Dunnett’s multiple comparisons test (*P < 0.05, **P < 0.01, ***P < 0.001, ****P < 0.0001)

The periplasmic binding proteins—*mppA*, the L-alanyl-g-D-glutamyl-meso-diaminopimelate binding protein, or *dppA*, the dipeptide binding protein—actively import ALA through an interaction with the dipeptide inner membrane ATP-binding cassette transporter, *DppBCDF*, in *E. coli* [29]. We theorized that inactivation of *dppA* and/or *mppA* genes would reduce ALA assimilation and therefore increase ALA accumulation in the medium. As expected, inactivation of *dppA* improved ALA production but *mppA* did not (Table 3). To further improve ALA production, we used the *E. coli* D4:FYAB strain to perform *dppA* and *mppA* gene knockouts individually or jointly. We found that mutant strain, *E. coli* D5:FYABD, showed the highest production of ALA which was 2.42 fold greater than D0 (Table 3).

### Growth characteristics of mutant strains and selection of optimal mutant *E. coli* strain

After knocking out these genes, growth of mutant strains in different media were examined to assess whether CRISPR/Cas9-mediated gene knock-out affected metabolic characteristics of the bacteria. These strains all harbored pET-ALA-LAA for producing ALA.

**Fig. 5:**
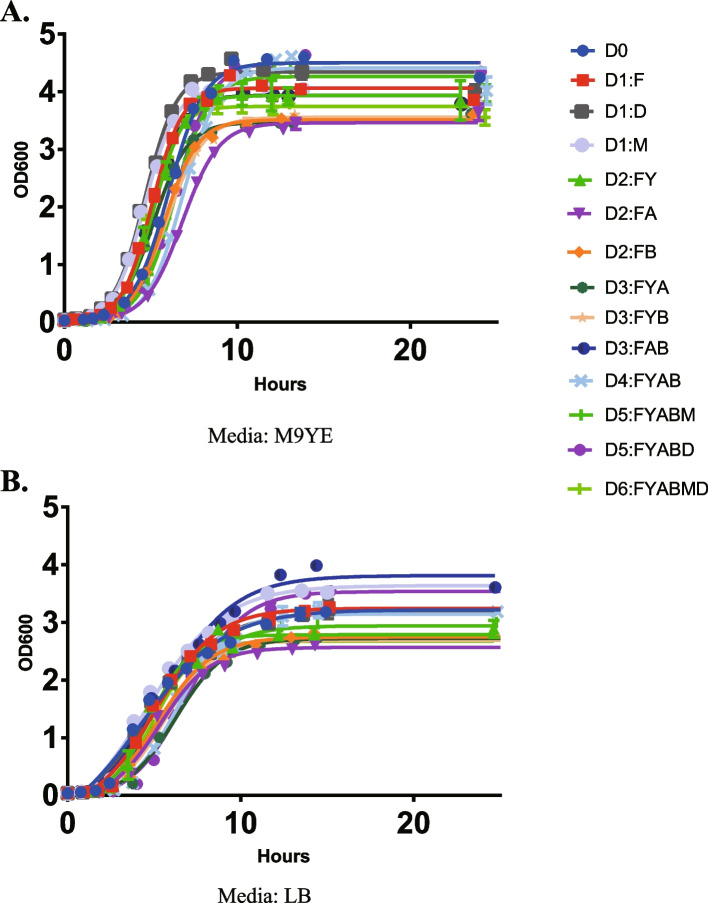
Growth characteristic of mutant *E. coli* strains in different medium. Cells were cultured in M9YE (**A**) or LB (**B**) medium with 50 μg/mL Kanamycin. Bacteria contained pET28b-ALA-LAA. Data are means of three replicates

These mutant strains were cultured in M9YE medium (see ‘‘Section Materials and Methods’’) or standard LB medium (Fig. 5). Growth rate among these strains in LB medium ranged from 0.25 to 0.3 (Table 4). When cultured in M9YE medium, growth rate of these strains varied from 0.3 to 0.5. Two mutant strains (D2:FA and D3:FYB) showed significant change in growth rate when culture in M9YE medium. These results reveal that knockouts of some genes may lead to growth retardation of bacteria.

**Table 4:** Growth rate of these mutant strains in different medium

Strain	Growth Rate	Medium	Significant Summary
*E. coli* BW25113-T7 (D0)	0.246	LB	
*E. coli* D1:F	0.271	LB	ns
*E. coli* D1:D	0.257	LB	ns
*E. coli* D1:M	0.278	LB	ns
*E. coli* D2:FY	0.305	LB	ns
*E. coli* D2:FA	0.238	LB	ns
*E. coli* D2:FB	0.252	LB	ns
*E. coli* D3:FYA	0.225	LB	ns
*E. coli* D3:FYB	0.242	LB	ns
*E. coli* D3:FAB	0.297	LB	ns
*E. coli* D4:FYAB	0.265	LB	ns
*E. coli* D5:FYABD	0.271	LB	ns
*E. coli* D5:FYABM	0.249	LB	ns
*E. coli* D6:FYABMD	0.287	LB	ns
*E. coli* BW25113-T7 (D0)	0.429	M9YE	
*E. coli* D1:F	0.457	M9YE	ns
*E. coli* D1:D	0.510	M9YE	ns
*E. coli* D1:M	0.479	M9YE	ns
*E. coli* D2:FY	0.445	M9YE	ns
*E. coli* D2:FA	0.305	M9YE	**
*E. coli* D2:FB	0.343	M9YE	ns
*E. coli* D3:FYA	0.360	M9YE	ns
*E. coli* D3:FYB	0.335	M9YE	*
*E. coli* D3:FAB	0.430	M9YE	ns
*E. coli* D4:FYAB	0.404	M9YE	ns
*E. coli* D5:FYABD	0.404	M9YE	ns
*E. coli* D5:FYABM	0.406	M9YE	ns
*E. coli* D6:FYABMD	0.443	M9YE	ns

*P* values were calculated using Dunnett’s multiple comparisons test (*P < 0.05, **P < 0.01)

**Fig. 6:**
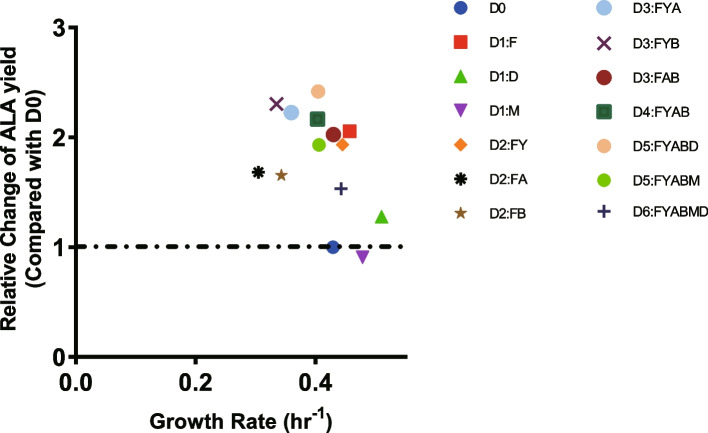
The results of all mutant strains with respect to ALA production and growth rates. The ALA production and growth rates of the control D0 strain harboring pET28b-ALA-LAA are also indicated for comparison

We easily selected D5:FYABD as the optimal mutant strain for ALA production based on its growth rate and ALA production (Fig. 6). The ALA production was increased 2.42-fold with similar growth rate when compared with the original-type (D0).

### Increasing ALA accumulation via C4 pathway

**Fig. 7:**
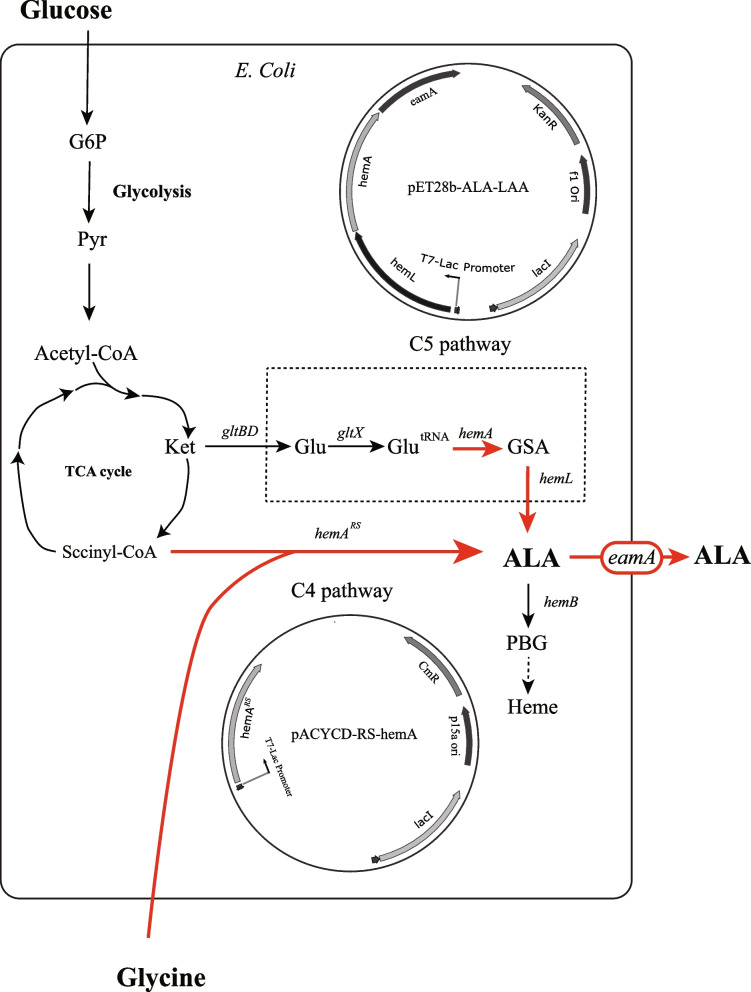
Schematic presentation ALA of production in *E. coli* via both C4 and C5 pathways. Glycine was added to the culture medium as a substrate for the C4 pathway. G6P, glucose-6-phosphate; Pyr, pyruvate; Ket, α-ketoglutarate; Glu, glutamate; Glu^tRNA^, Glutamyl-tRNA; GSA, glutamate 1-semialdehyde aminotransferase; ALA, 5-aminolevulinic acid; PBG, porphobilinogen

**Fig. 8:**
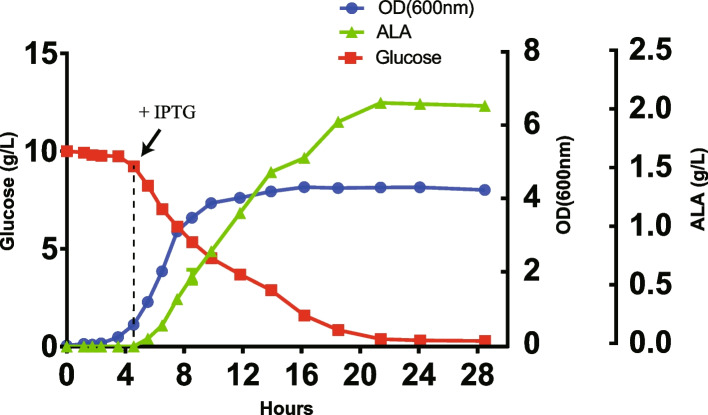
Fermentation of ALA in *E. coli* D5:FYABD-RSA. A 1% (v/v) inoculum from an overnight culture for 12 h was used. 10 g/L glucose and 2 g/L glycine were added initially. 0.1 mM IPTG was added as indicated. During the fermentation, the pH was controlled optimally at 6.5 ± 0.3

In *E. coli*, primary ALA biosynthesis is through the C5 pathway. To further increase accumulation of ALA, we tried to synergistically produce ALA via the C4 pathway. The photosynthetic bacterium, *Rhodobacter sphaeroides*, can accumulate ALA under certain conditions or after mutagenesis [30–32]. Through metabolic engineering, recombinant *E. coli* was also able to produce ALA from the C4 pathway through biotransformation [33]. In this aspect, the gene encoding for ALA synthase from *R. sphaeroides* was introduced into *E. coli* through genetic engineering [33]. Biosynthesis of glycine and succinyl-CoA are regulated in *E. coli*. Consequently, glycine and succinate (the precursor of succinyl-CoA) must be added to the culture medium artificially to provide sufficient substrates for enhanced ALA biosynthesis. In the current study, we overexpressed a modified heterologous *hemA* from *R. sphaeroides* using the T7 Expression System (Fig. 7). The amino acid sequence of *hemA*^*RS*^ was modified for better expression in *E. coli*. Glycine (2 g/L) was added to the M9YE medium as a substrate for the C4 pathway. These modifications increased ALA accumulation from 1602.72 mg/L with *E. coli* D5:FYABD to 2099.7 mg/L for the mutant strain containing the *hemA*^*RS*^ gene (Table 2 and Fig. 3B). After 24 h cultivation, a yield of 0.210 g ALA per g glucose was achieved (Fig. 8).

## Discussion

Metabolic engineering provides a powerful tool for regulating metabolic pathway towards accumulation of desired compounds [34]. Many bio-based products are now produced in *E. coli* through metabolic engineering or synthetic biology [17, 35–37]. In the present study, we developed a metabolic strategy to produce ALA directly from glucose and constructed a well-defined genetically-engineered *E. coli* strain based on known metabolic and regulatory information and CRISPR-Cas9 mediated gene knock-outs. After systematic metabolic engineering of *E. coli*, we were able to achieve a high yield of 0.210 g of ALA per gram of glucose. More importantly, the engineered strain developed in this study can be further improved because all of the modifications are clearly defined.

We first constructed the ALA-producing *E. coli* base strain by amplifying three key genes. *HemA* encodes Glutamyl-tRNA reductase, which catalyzes the first step of porphyrin biosynthesis [38]. Glutamate-1-semialdehyde 2,1-aminomutase (*hemL*) catalyzes the pyridoxal 5'-phosphate-dependent transfer of the amino group from C2 of glutamate-1-semialdehyde (GSA) to C1, thereby forming ALA [39]. Synergistic functions of *hemA* and *hemL* efficiently converted glutamate to ALA. The *eamA* gene encoded an integral membrane protein which is implicated in exporting metabolites of the cysteine pathway [40]. Deletion of *eamA* in *E. coli* strain BL21 is associated with a decline in extracellular 5-aminolevulinate concentration [41] which suggests that *eamA* might be a transporter involved in export cysteine pathway metabolites. We overexpressed *hemA*, *hemL* and *eamA* genes using T7 system in *E. coli* BW25113-T7 and achieved a yield of 0.066 g of ALA per gram of glucose. Then several genes encoding those enzymes directly involved in Glutamate and L-glutamyl-tRNA biosynthesis were amplified. However, this modification did not increase accumulation of ALA. This result was similar to previous research by Kang [17]. In their study, amplifying *gltX* genes decreased ALA accumulation. Our result also suggested biosynthesis of L-glutamate and conversion of L-glutamate to Glu^tRNA^ are not rate-limiting steps in ALA biosynthesis. Overexpression of *gltX*, *gltB* and *gltD* genes may increase metabolic stress on cells which may lead to growth retardation and decreased ALA accumulation.

Further improvement of the ALA-producing strain was achieved to modify metabolic pathway rationally by using the metabolic and regulatory information available in the literature (Fig. 1). In *E. coli*, biosynthesis of ALA via the C5 pathway is tightly regulated by the end product heme [16]. Synthesis of heme leads to an endogenous depletion of ALA. Additionally, heme also makes *hemA* unstable which inhibits accumulation of ALA [42]. Therefore, we expected that down-regulation of heme biosynthesis might lead to high ALA production. However, knock-outs of most genes encoding enzymes directly involved in heme biosynthesis (*hemBCDEGH*) lead to severe growth limitation or even death. Oxidative decarboxylation of coproporphyrinogen III to protoporphyrinogen IX is catalyzed by two enzymes in *E. coli*. Under aerobic conditions, the *hemF* gene product is active. Under anaerobic conditions, *HemN* catalyzes the coproporphyrinogen III dehydrogenase reaction. Hence, we believed that knock-out of *hemF* will ultimately restrict biosynthesis of heme. This belief was confirmed with the observation that the mutant strain *E. coli* D1:F increased ALA yield from 662.25 to 1361.22 mg/L.

Our next step was to knock out the genes responsible for major competing pathways based on published metabolic and regulatory information (Fig. 1). The major competing pathways, including nitrogen metabolism, glutathione metabolism and butanoate metabolism, were suppressed in the present study. In *E. coli*, there are two pathways to synthesize glutamate from ammonia [19]. The pathway catalyzed by glutamate dehydrogenase (encoded by *gdhA*) directly from ammonia, α-ketoglutarate, and NADPH [26]. The other is a cyclic and ATP-driven pathway (Shown in Fig. 2) and is catalyzed by glutamate synthase (encoded by *gltBD*). Each turn of which utilizes one molecule each of ammonia, ATP, NADPH, and α-ketoglutarate to produce one molecule of glutamate [19]. When culture in our modified minimal medium (M9YE) which does not provide a high level of ammonia, the ATP-driven pathway functions (Fig. 1). As the reaction catalyzed by *gdhA* is reversible, we assumed that down-regulation of *gdhA* catalyzed reversible reaction is believed to reduce endogenous loss of L-glutamate and led to a raise of ALA production. The *ybdK* (encoding putative glutamate—cysteine ligase 2) and *gadB* (encoding glutamate decarboxylase B) genes were also knocked out to increase L-glutamate availability for ALA synthesis. In our study, all triple mutant strains (D3:FYA, D3:FYB and D3:FAB) yielded more ALA than double mutant strains (D2:FY, D2:FA and D2:FB). We propose that synergistical effects of knocking out these three genes (*ybdK*, *gadB* and *gdhA*) improves ALA yields because of the increased flux through L-glutamate. These observations led us to select mutant strain D4:FYAB for further modification even though this strain did not produce the highest yield of ALA.

To avoid ALA accumulation inside cells and to produce more ALA in the medium, inactivation of *mppA* and/or *dppA* genes was carried out. Cultivation results showed that inactivation of *dppA* (D1:D) improved ALA production while inactivation of *mppA* (D1:M) did not. The D1:D strain harboring pET28b-ALA-LAA accumulated 27.9% more ALA than the D0 strain did (Table 3). Further knockout of *dppA* and/or *mppA* genes in strain D4:FYAB showed a similar result. The additional knockout of the *mppA* gene in D4:FYAB did not increase ALA production. Double mutant of *dppA* and *mppA* in D4:FYAB strain even showed a 29.2% decrease in ALA production. These results suggest that silencing the *dppA* gene is more beneficial for ALA biosynthesis than knocking out the *mppA* gene.

According to metabolic and regulatory information available in the literature, we identified a mutant strain that allowed elevated ALA production with an acceptable growth rate. In the D5:FYABD strain, heme biosynthesis was limited and flux through glutamate was increased. Deletion of the *dppA* gene reduced ALA assimilation by *E. coli* which allowed increased ALA accumulation in the medium. The D5:FYABD strain harboring pET-ALA-LAA showed a 141.8% increase in ALA production compared with the D0 strain harboring the same plasmids. These results suggest that beneficial effects on ALA production of flux redistribution achieved in D5:FYABD strain could be further strengthened by engineering regulatory and export functions.

There are two major pathways described for ALA biosynthesis in living organisms [13]. We successfully measured production of ALA via the C5 pathway in this study and tried to develop a strategy to improve ALA production in recombinant *E. coli* via the C4 pathway. The ALA synthase from *R. sphaeroides* was introduced into *E. coli* to produce ALA from the C4 pathway. This modified *hemA*^*RS*^ was able to work synergistically with native *hemA* and *hemL* from *E. coli* (Fig. 7). In *R. sphaeroides*, glycine and succinyl-CoA can be catalyzed to form ALA by *hemA*. Since biosynthesis of glycine and succinyl-CoA was also regulated in *E. coli*, we added glycine in the medium artificially to provide more substrates for ALA biosynthesis. Through this strategy, ALA production in D5:FYABD was increased from 1601.7 mg/L to 2099.7 mg/L, a 31.1% increase in ALA accumulation.

## Conclusion

In summary, rational metabolic engineering based on known metabolic and regulatory information, and co-expression of native key genes and modified heterologous gene, allowed development of an *E. coli* strain capable of efficiently producing ALA. An impressively high yield of 0.210 g of ALA per gram of glucose could be achieved using a metabolically engineered strain in flask culture. Further optimization of the fermentation process could improve ALA production to even higher levels. The approaches described in this study can also be applied more broadly to develop strains for efficient production of other metabolites.

## Methods and materials

### *E. coli* strains

Molecular cloning and manipulation of plasmids were done with *E. coli* DH5α (TransGen, Beijing). BW25113-T7 strains were used for CRISPR/Cas9-induced Double Strain Break and recombination. All *E. coli* strains were cultured routinely in standard LB medium when not mentioned otherwise.

### Growth conditions

LB medium (10 g/L tryptone, 5 g/L yeast extract and 10 g/L NaCl, pH 7.2) was used in all DNA manipulations. During cultivation and fermentation, the modified M9 medium (M9YE) was used that contained 1 g/L NH_4_Cl, 0.5 g/L NaCl, 3 g/L KH_2_PO_4_, 17.1 g/L Na_2_HPO_4_·12H_2_O, 2 mM MgSO_4,_ 0.1 mM CaCl2, 2 g/L yeast extract and 10 g/L glucose. Glycine (2 g/L) was added as indicated to serve as the substrate for the C4 pathway. Ampicillin (100 mg/mL), chloramphenicol (25 mg/mL) and kanamycin (50 mg/mL) were added to provide selective pressure for *E. coli* during cultivation when necessary. To induce expression of plasmid-borne genes, Isopropyl-β-D-thiogalactopyranoside (IPTG) was added to cultures which resulted in a final concentration of 0.1 mM. Considering cell growth and ALA stability, the pH was measured by a glass electrode and controlled at 6.5 ± 0.3 with 4 M NaOH.

### Selection of integration site and design of homologous recombination

The function and detailed message of the *hemF* gene (and other genes) was verified in NCBI and BioCyc Database. Sequence of the *hemF* gene (and other genes) in the BW25113-T7 genome was confirmed in NCBI. As the recognition site for sgRNA, N20 site directs the Cas9 protein to enable site-specific induction of a DSB. The N20 site was found by BROAD international design tool, which is available at: http://www.broadinstitute.org/rnai/public/analysis-tools/sgrna-design.

### Plasmid construction for CRISPR and preparation of linear donor dsDNA by PCR

All primer pairs we designed for gene cloning and intermediate plasmid construction are listed in Table S2. Plasmid pCas (Fig. S1A) and pTarget were prepared in our laboratory. Plasmid pTarget-gene was constructed by Reverse-PCR and T4-Ligation (T4 DNA Ligase, NEB, England) to replace the N20 fragment (Fig. S1B) with specific primers (Table S2). Plasmid pACYCD-gene and pACYCD-Donor-Gene were both constructed for preparing Donor DNA (Fig. S2) by In-Fusion® HD Cloning Kit (Takara, Japan). Donor DNA was cloned from pACYCD-Donor-Gene by Hi-Fi PCR (Phusion® High-Fidelity PCR Master Mix, NEB, England). All plasmids used in this research are listed in Table 1.

### Electroporation, cell recovery, and plating

For transformation, the plasmid or linear DNA were electroporated into competent cells in the pre-chilled cuvette (0.1 cm) using Bio-Rad MicroPulser (1.8 kV, time constant > 5.0 ms). For selection, 25 μg/mL chloramphenicol (Chl) or 50 μg/mL kanamycin (Kan) were used alone or in combination. For induction of λ-Red proteins and lac operator, 1 mM arabinose and 1 mM IPTG were used.

To prepare cells harboring pCas, cells cultured at 37℃ (OD_600_ = 0.45–0.55) were made competent, mixed with pCas (100 ng) and subjected to electroporation, after which the cells were recovered in SOC medium (1 mL) for 1 h at 30℃, plated onto the Kan plate, and cultured at 30 ℃ for 18–24 h.

For CRISPR/Cas9-mediated homologous recombination, cells harboring pCas were cultured at 30℃ in medium containing Kan and Arabinose and made competent. After co-electroporation of Donor DNA (400 ng) with pTarget-Gene (100 ng), cells were recovered in SOC (1 mL) medium for 1 h at 30℃, plated onto Chl/Kan plate, and cultured at 30℃ for 18–24 h.

For elimination of pTarget-Gene, cells harboring both pCas and pTarget were cultured at 30℃ in medium containing Kan and IPTG for 2 h. Cells were plated onto Kan plates and cultured at 30℃ for 18–24 h.

For elimination of pCas, cells harboring pCas were cultured at 37℃ in the medium without any antibiotic for 12–16 h. Then the cells were plated onto non-antibiotic plates and cultured at 37℃ for 12–16 h.

### Confirmation of CRISPR/Cas9-mediated gene knock-in in BW25113

The targeted genetic modifications were rationally engineered by CRISPR/Cas9 in *E. coli* BW25113-T7. The six targeted genes (*hemF*, *ybdK*, *gadB*, *gdhA*, *dppA* and *mppA*) were cloned from *E. coli* BW25113-T7 to prepare for Donor DNA. The process of *hemF* gene knock-out is shown here as example. The map of *hemF* gene knock-out in BW25113-T7 in ideal condition is shown in Fig. S3. The homologous left arm (HRL) and the homologous right arm (HRR) were set near site of N20. Both homologous arms were approximately 400 bp, which could have a high efficiency of recombination [43]. After CRISPR/Cas 9 mediated gene knock-out, it would make a deletion of 100 bp DNA in targeted gene.

To make CRISPR/Cas9-mediated homologous recombination in BW25113-T7, we electroporated pCas (encoding both Cas and λ-Red proteins) into *E. coli* BW25113-T7, followed by Arabinose (Ara) induction of pCas-encoded λ-Red proteins Gam, Bet and Exo. After preparing competent cells, pTarget-gene (such as pTarget-hemF) and Donor DNA were co-electroporated into cells (Fig. S4).

To verify deletion in target locus, all mutated bacterial strains were selected for colony PCR (Fig. S5). The primer pairs for colony PCR are shown in Table S2. Characteristics of those mutant strains are shown in Table 1.

### Growth of bacteria in different medium

All *E. coli* strains were grown at 37℃ in conical flasks (250 mL) containing M9YE medium or LB medium with Kanamycin (50 μg/mL). Growth was measured by monitoring optical density at 600 nm (OD_600_) using a spectrophotometer.

The growth rate was fitted with Sigmoidal-4PL curves. The OD of the stationary phase (OD_sp_) and the time required to reach it are calculated from this fitted curve. The ratio of the OD_sp_ to the time required to reach it was set as growth rate (hr-1) of the strain in medium.

### Analytical procedures

Flask cultivations were carried out in 100 mL conical flasks supplied with 30 mL modified minimal medium at 37℃ with agitation of 200 rpm. A 1% (v/v) inoculum from an overnight culture (12) was used. Cells were cultured in M9YE at 37℃ until OD_600_ reached 0.7. Then IPTG with a final concentration of 0.1 mM was added to the induced group. To analyze ALA production, culture (30 mL) after inducing for 24 h was centrifuged (12,000 × g for 2 min at 4℃). The supernatant was analyzed for extracellular ALA concentration. ALA concentration was analyzed using modified Ehrlich’s reagent [44]. Specifically, standard or sample (2 ml after diluted) was mixed with 1 ml 1.0 M sodium acetate (pH 4.6) in a cuvette, and 0.5 ml acetylacetone (2,4-pentanedione) was added to each cuvette. Then the mixtures were heated to 100 ℃ for 15 min. After cooling for 15 min, the reaction mixture (1 ml) and freshly prepared modified Ehrlich's reagent (1 ml) were mixed together. After 30 min, the absorbance at 554 nm was measured. Standard plot for ALA measurement is shown in Fig. S6.

For analyzing glucose, 1 mL of culture was centrifuged (12,000 g for 2 min at 4 ℃) and the supernatant was then filtered through a 0.22 mm syringe filter for analysis. The HPLC system was equipped with a cation exchange column (HPX-87 H, BioRad Labs), and a differential refractive index (RI) detector (Shimadzu RID-10 A). A 0.5 mL/min mobile phase using 5 mM H_2_SO_4_ solution was applied to the column. The column was operated at 65 ℃.

### Data analysis

Data for ALA production of were subjected to analysis of variance (ANOVA) by GraphPad Prism (version 7.00). Error bars indicate standard error of the mean (SEM). *P* values were calculated using Dunnett's multiple comparisons test (*P < 0.05, **P < 0.01, ***P < 0.001, ****P < 0.0001). The mean of each column was compared with the mean of a control column. BW25113-T7 (D0) was set as control column.

## Supplementary Information

**Additional file 1: Fig S1.** Map of plasmids which were constructed for CRISPR. A) Map of pCas, which harbored the temperature sensitive oriR101 with repA101ts, kanamycin resistance gene, the λ-Red operon encoding Gam, Bet, and Exo proteins under the control of arabinose-inducible promoter ParaB, *S. pyogenes*-derived cas9 driven by endogenous promoters and sgRNA guided to ori-p15a which is under the control of lac operator. B) Map of pTarget-gene, which harbored Chloramphenicol resistance, ori-p15a and sgRNA guided to *E. coli* BW25113-T7 targeted gene.

**Additional file 2: Fig S2.** Construct of intermediate cloning vectors for preparing Donor DNA. (A) Fragment A cloned from BW25113-T7 was concatenated to pACYCD-Blank to assemble pACYCD-gene. (B) Reverse-PCR to constructed pACYCD-Donor. (C) The map of Donor DNA, which contains HRL and HRR.

**Additional file 3: Fig S3.** Gene map for targeted Cas9-mediated gene Knock-In. (A) The knock-out site of* hemF* in BW25113-T7. (B) map of *hemF* knock-out in ideal condition and the location of PCR product (899 bp) for sequencing.

**Additional file 4: Fig S4.** Schematic illustration of DSB induction and homologous recombination. After preparing competent cells, the pTarget-gene and Donor DNA which harbored homology arms (HRR and HRL) that targeted a chromosomal locus spanning the middle of targeted gene and the DSB site were electroporated into cells.

**Additional file 5: Fig S5.** Detection of successful gene knock-outs of BW25113-T7.

**Additional file 6: Fig S6.** Standard Plot for ALA measurement. x: the absorbance at 554 nm; y: ALA concentration of sample after diluted (mg/L).

**Additional file 7: Table S1.** Growth rate of strain expressing various related genes in different medium.

**Additional file 8: Table S2.** Primers for Plasmid constructions and Testing.

## Data Availability

The majority of data generated or analyzed during this study are included in this published article or in the supplementary information. Data not shown in this manuscript are available upon request from the corresponding author.

## Abbreviations

*E. coli*: *Escherichia coli*
ALA: 5-Aminolevulinic Acid
GSA: Glutamate-1-semialdehyde
GluRS: Glutamate-tRNA ligase
IPTG: Isopropyl-β-D-thiogalactopyranoside
Kan^r^: Kanamycin resistance gene
Chl^r^: Chloramphenicol resistance
sgRNA: Small-guide RNA
HRL: Homologous left arm
HRR: Homologous right arm
