# Supplementary material for: Metabolic engineering of Escherichia coli BW25113 for the production of 5-Aminolevulinic Acid based on CRISPR/Cas9 mediated gene knockout and metabolic pathway modification
Source: J Biol Eng. 2022 Oct 13;16:26. doi: 10.1186/s13036-022-00307-7 (PMC9563957; doi:10.1186/s13036-022-00307-7)
Supplement: Supplementary file 7 — Additional file 7: Table S1. Growth rate of strain expressing various related genes in different medium. [file 13036_2022_307_MOESM7_ESM.pdf]

Table S1. Growth rate of strain expressing various related genes in different medium

Strain	Plasmid	Expressed genes	Growth Rate	Medium
E. coli BW25113-T7	pET-ALA-LAA	*hemA, hemL, eamA*	0.249	LB
E. coli XD	pET-ALA-LAA + pUC-gltX-gltD	*hemA, hemL, eamA, gtlX, gltD*	0.203	LB
E. coli BX	pET-ALA-LAA + pUC-gltB-gltX	*hemA, hemL, eamA, gltB, gltX*	0.195	LB
E. coli BD	pET-ALA-LAA + pUC-gltB-gltD	*hemA, hemL, eamA, gltB, gltD*	0.180	LB
E. coli BDX	pET-ALA-LAA + pUC-gltB-gltX-gltD	*hemA, hemL, eamA, gltB, gltD, gltX*	0.177	LB
E. coli BW25113-T7	pET-ALA-LAA	*hemA, hemL, eamA*	0.442	M9YE
E. coli XD	pET-ALA-LAA + pUC-gltX-gltD	*hemA, hemL, eamA, gtlX, gltD*	0.251	M9YE
E. coli BX	pET-ALA-LAA + pUC-gltB-gltX	*hemA, hemL, eamA, gltB, gltX*	0.281	M9YE
E. coli BD	pET-ALA-LAA + pUC-gltB-gltD	*hemA, hemL, eamA, gltB, gltD*	0.242	M9YE
E. coli BDX	pET-ALA-LAA + pUC-gltB-gltX-gltD	*hemA, hemL, eamA, gltB, gltD, gltX*	0.190	M9YE
