# Supplementary material for: Metabolic engineering of Escherichia coli BW25113 for the production of 5-Aminolevulinic Acid based on CRISPR/Cas9 mediated gene knockout and metabolic pathway modification
Source: J Biol Eng. 2022 Oct 13;16:26. doi: 10.1186/s13036-022-00307-7 (PMC9563957; doi:10.1186/s13036-022-00307-7)
Supplement: Supplementary file 8 — Additional file 8: Table S2. Primers for Plasmid constructions and Testing. [file 13036_2022_307_MOESM8_ESM.pdf]

Primer	Sequences (5'to3')	Base Number
ACYCD-hemF-F	gcgagcgcaatttccctgccattcatccgcttattatc	38
ACYCD-hemF-R	tgcggctgtgcagttcctggcgttacccaacttaa	35
HRL-hemF-F	aactgcacagccgcaacac	19
HRR-hemF-R	ggaaattgcgctcgcgct	18
HRL-hemF-R	cccgtatgttcccaccagc	19
HRR-hemF-F	gcctgcctgttcgaaaacac	20
N20argI-s	gttttagagctagaaatagcaagttaaaat	30
N20-hemF	CTCATCGCCCGGAACTTGCCactagtattatacctaggact	43
Test-hemF-F	tgacgctcggctcgcatatt	20
Test-hemF-R	ccgcgatcccagaccagatt	20
HRL-gdhA-F	gcgacccgaatcaaaccgag	20
HRR-gdhA-R	tagagaccacggctgttggcc	21
UC-gdhA-R	gtttgattcgggtcgcctgacgggcttgtctgctc	35
UC-gdhA-F	cagccgtggtctctactgtcgtgccagctgcatta	35
HRR-gdhA-F	gcggggatgatgaaaaagctctc	23
N20-gdhA	acgttccggcaggtgatatcactagtattatacctaggactga	43
HRL-gdhA-R	cctggcaaaaacgcatcact	20
test-gdhA-R	gtcaacatccagttcattctgggtg	25
test-gdhA-F	tctctggagtcattcctcaaccatg	25
HRL-dppA-F	ttttggcctgcacgccgacttt	22
HRR-dppA-R	ggtgtgaagtggcacgacaataaag	25
UC-dppA-R	gcgtgcaggccaaaactgacgggcttgtctgctc	34
UC-dppA-F	gtgccacttcacaccctgtcgtgccagctgcatta	35
HRR-dppA-F	ttggtgccccagtagccatc	20
N20-dppA	ctgacgcttccgtgcgttacactagtattatacctaggactga	43
HRL-dppA-R	acccggcagatatcgctcg	19
Test-dppA-R	gaagtcagcgaagacggtaaaacc	24
Test-dppA-F	cgcttgaggtactcacccca	20
HRL-mppA-F	atgtgctttaccgcattttggagac	25
HRR-mppA-R	agtggctgcggattcctgat	20
UC-mppA-R	tgcggtaaagcacatctgacgggcttgtctgctc	34
UC-mppA-F	gaatccgcagccactctgtcgtgccagctgcatta	35
HRR-mppA-F	aacgtctggtggacccaaaaac	22
N20-mppA	cccattttgcgttatcgcgcactagtattatacctaggactga	43
HRL-mppA-R	gcaacgccggggacaatctc	20
Test-mppA-R	aacctgccccggaatatcct	20
Test-mppA-F	gaaaacgggtttgaacgtgcg	21
